# Extended Kalman Filter for Estimation of Parameters in Nonlinear State-Space Models of Biochemical Networks

**DOI:** 10.1371/journal.pone.0003758

**Published:** 2008-11-19

**Authors:** Xiaodian Sun, Li Jin, Momiao Xiong

**Affiliations:** 1 Laboratory of Theoretical Systems Biology and Center for Evolutionary Biology, School of Life Science and Institute for Biomedical Sciences, Fudan University, Shanghai, China; 2 CAS-MPG Partner Institute of Computational Biology, SIBS, CAS, Shanghai, China; 3 Human Genetics Center, University of Texas Health Science Center at Houston, Houston, Texas, United States of America; IBM Thomas J. Watson Research Center, United States of America

## Abstract

It is system dynamics that determines the function of cells, tissues and organisms. To develop mathematical models and estimate their parameters are an essential issue for studying dynamic behaviors of biological systems which include metabolic networks, genetic regulatory networks and signal transduction pathways, under perturbation of external stimuli. In general, biological dynamic systems are partially observed. Therefore, a natural way to model dynamic biological systems is to employ nonlinear state-space equations. Although statistical methods for parameter estimation of linear models in biological dynamic systems have been developed intensively in the recent years, the estimation of both states and parameters of nonlinear dynamic systems remains a challenging task. In this report, we apply extended Kalman Filter (EKF) to the estimation of both states and parameters of nonlinear state-space models. To evaluate the performance of the EKF for parameter estimation, we apply the EKF to a simulation dataset and two real datasets: JAK-STAT signal transduction pathway and Ras/Raf/MEK/ERK signaling transduction pathways datasets. The preliminary results show that EKF can accurately estimate the parameters and predict states in nonlinear state-space equations for modeling dynamic biochemical networks.

## Introduction

Cells are complex interconnected web of dynamic systems. They involve metabolites, genes and proteins which are organized into different biochemical reaction networks: metabolic, signal transduction and gene regulation networks, and protein interaction networks which form complex biological systems [1].These biochemical reaction networks control cell proliferation, differentiation, and survival [2]. To unravel the rules that govern behavior of biological systems is the focus of molecular biology researches. To gain a deep understanding about the biological systems requires modeling of biochemical reaction networks. Simple empirical description of biochemical reaction networks is insufficient for discovery of the general principles underlying biological process and prediction of dynamic response of biological networks to drug interventions or environmental perturbation [3]. The inherent properties of complex biochemical reaction networks are hard to elucidate by intuition [4]. Mathematical and computational modeling of biochemical reaction networks can comprehensively integrate experimental knowledge into forming and testing hypotheses and help to gain into system level understanding of biochemical networks, which will not been seen if the components of biochemical networks are separately studied. Therefore, developing mathematical models of biological systems holds a key to understanding and predicting the dynamic behaviors of the biological systems under perturbation of external stimuli and hence a major task of systems biology and is the keystones of systems biology [5].

Two basic types of approaches: bottom-up approach and top-down approach have been widely used in mathematical modeling of biochemical reaction networks [6]. Bottom-up approach usually assumes the mechanistic kinetic models. A full understanding of biochemical reaction networks requires quantitative information about the structure of the networks, kinetic laws and the concentrations of metabolites, enzymes and proteins [7]. The kinetic models allow us to test hypotheses, investigate the fundamental design principles of cell functions, and predict the dynamic changes of concentration of metabolites and proteins [8]. The kinetic models explicitly incorporate prior knowledge about biochemical mechanism underlying biological processes into the model and hence can serve as the basis for studying the effects of direct intervention for improving desired properties of biological systems. Top-down approach assumes “black-boxes” models about the molecular organization of biochemical networks and quantifies the input and output relations in biochemical networks. The kinetic models are undoubtedly a major tool for investigation of biochemical networks [9].

A great challenge in kinetic modeling of biochemical networks is to identify the structure of the networks and estimate kinetic parameters in the model [10]. Since most kinetic models of biochemical networks are nonlinear it is extremely difficult to identify the structure of the networks by computational methods. They are often determined by experiments. We are mainly concerned with estimation of kinetic parameters in this report. It is increasingly recognized that it is dynamics of the systems that determines the function of cells, tissues and organisms. Successful modeling which can unravel inherent dynamic properties of biochemical networks requires time-course quantitative measurements of metabolites, enzymes and proteins, although these measurements are still difficult to obtain [11]. A general framework for parameter estimation is to estimate the parameters in the mathematical model of the biochemical network, given time-course experimental data [12]. Parameter estimation in nonlinear dynamic systems is extremely important, but also extremely difficult. Most current methods for parameter estimation, in principle, are to formulate the parameter estimation problem as a nonlinear optimization problem with differential-algebraic constraints that describe dynamics of biochemical networks. The objective function of the optimization is the discrepancy between model prediction, which are obtained from simulations using assumed model with estimated parameters, and the experimental data [13].Various deterministic and stochastic optimization methods have been used to solve the formulated nonlinear dynamic optimization problems [14]–[18].

However, nonlinear dynamic optimization approach to parameter estimation of biochemical networks has a number of limitations. First, computational cost for nonlinear dynamic optimization is very high. Second, although measurement errors can be incorporated into the observation equations, it is difficult to integrate system noise into rate equations (or system equations). Third, due to the high nonlinearity and nature of dynamic constraints, nonlinear dynamic optimizations of the parameter estimation of biochemical networks are often multimodal. Therefore, their solutions may not reach global optimum. They often converge to a local optimum [14].

To overcome these limitations, parameter estimation for rate equation models of biochemical networks can be formulated as parameter estimation for nonlinear state-space models that consist of two types of variables: state variables (hidden variables) and observed variables, and two types of equations: system equations and observation equations [19]. Essential nature of the rate equation models of biochemical networks is that some variables in the models are not observable. These unobserved variables can be taken as state variables. Nonlinear rate equations that are ordinary differential equations describe evolution of dynamics of concentrations of metabolites, enzymes and proteins over time. The observed variables are functions of the states of the dynamic system of biochemical networks. Estimation problems in nonlinear state-space models are addressed mainly within a probability framework. In other words, the complete solution to the parameter estimation problem is determined by the conditional probability density function of the states *X*, given the observed data *Y*, *P_θ_*(*X*|*Y*), where *θ* is parameters. Due to its extreme complexity, we must resort to approximation techniques for the solution to the parameter estimation in nonlinear dynamic systems. Most popular approaches to approximation are extended Kalman Filter [20]–[24] and sequential Monte Carlo methods [25]–[29], [30]–[32]. Extended Kalman filter (EKF) is to recursively approximate nonlinear model by a linear model and then use the traditional Kalman filter for the linearized model. The EKF for parameter estimation has a number of advantages. First, in its update rule, the EKF only use the mean and covariance of the state. The EKF is simple and computationally fast. Second, the EKF has close connection with the state-space theory. Third, the EKF has a unified formulation for both single variable and multivariable problems.

Purpose of this report is to use nonlinear state-space models as a general framework for investigation of dynamics of biochemical networks and formulate the estimation of parameters in biochemical networks as a recursive nonlinear state estimation problem. Since the EKF can jointly estimate both parameters and hidden states of the nonlinear dynamic systems, the EKF will be employed to solve the recursive nonlinear state estimation problem. To evaluate its performance, the EKF will be applied to the real JAK-STAT and Ras/Raf/MEK/ERK signaling transduction pathway data.

## Methods

### Kinetic Models for JAK/STAT Signal Transduction Pathway

**Figure 1:**
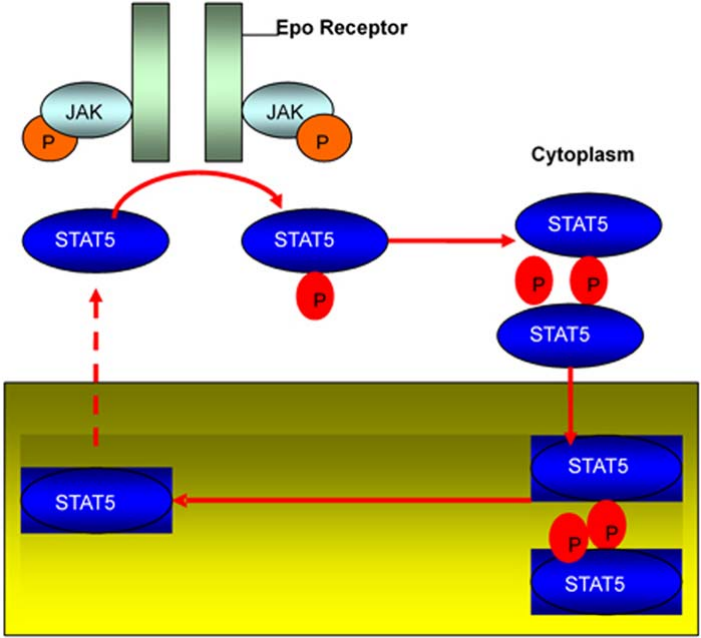
Scheme of the JAK-STAT pathway.

JAK/STAT which is initiated by cytokines is an important signal transduction pathway in regulating immune response [4], [33]. JAKs (Janus kinases) represent a family of non-receptor tyrosine kinases. STATs (signal transducers and activators of transcription) consist of a family of structurally and functionally related proteins [34]. As shown in Figure 1, binding of the ligand, the hormone erythroprotein (Epo) to the receptor activates the receptor associated Janus kinase (JAK) by phosphorylation, which in turn results in the recruitment of monomeric Stat5. Stat5 is then tyrosine-phosphorylated. The phosphorylated monomeric forms dimmers which migrate into the nucleus where they further bind to the promoter of target gene and initiate gene transcription. The dimerized Stat5 stops its active role by dedimerization and dephosphorylation. Dephosphorylated Stat5 is then exported to the cytoplasm.

Biochemical reactions for the JAK/STAT signal transduction pathway are given by
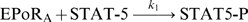


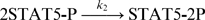


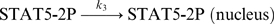


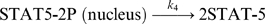
Let *x*_1_ be unphosphorylated monomeric STAT-5, *x*_2_ be phosphorylated monomeric STAT-5, *x*_3_ be phosphorylated dimeric STAT-5 in the cytoplasm and *x*_4_ be the phosphorylated dimeric in the nucleus. Let the rates of the reactions are given by
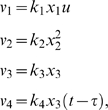
where *u* is the concentration of EpoR_A_ and *τ* denotes the delayed time.

The stoichiometrix, S, is
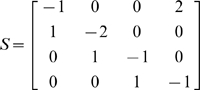
Let *x* = [*x*_1_, *x*_2_, *x*_3_, *x*_4_]*^T^* and *V*(*x*, *u*) = [*v*_1_, *v*_2_, *v*_3_, *v*_4_]*^T^*. The differential equations for the reactions of JAK-STAT signal transduction pathway is then expressed as
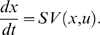
(1)The variables *x*_1_, *x*_2_, *x*_3_ and *x*_4_ are often not observed. The observed quantities are the concentrations of the phosphorylated STAT-5 in the cytoplasm and total unphosphorylated and phosphorylated STAT in the cytoplasm. The observed equations are then
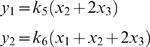
(2)


### Ras/Raf/MEK/ERK Pathway

**Figure 2:**
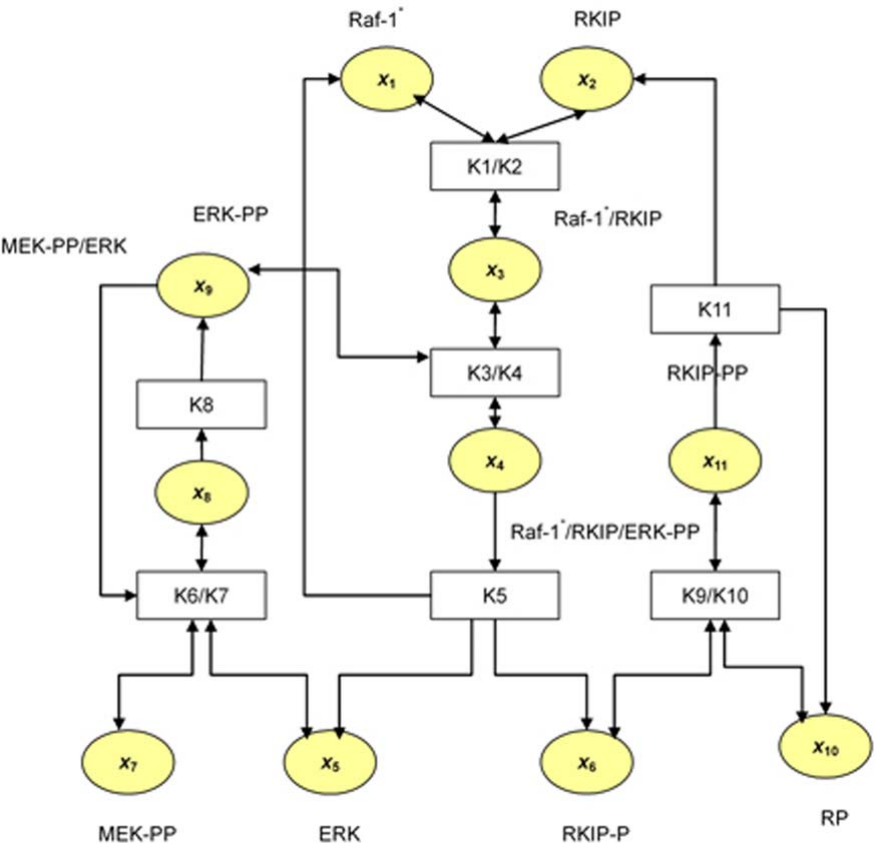
Scheme of the Ras/Raf/MEK/ERK Pathway.

The Ras/Raf/MEK(mitogen-activated protein kinase)/ERK (extracellular-signal-regulated kinase) pathway is a mitogen-activated protein kinase (MAPK) pathway, which is involved in proliferation, differentiation, survival and apoptosis processes [35]. The MAPK pathway consists of three kinases: a MAPK kinase kinase (MAPKKK), a MAPK kinase (MAPKK) and MAPK. There are six distinguishable MAPK modules that share structurally similar components, but perform specific biological tasks. In Ras/Raf/MEK/ERK pathway (Figure 2), Ras can be treated as a G-protein, Raf as MAPKKK, MEK as MAPKK and ERK as MAPK. Ras and Raf are proto-oncogenes. Growth factor receptors activate the G-protein Ras, which in turn binds to and activates the Raf-1 kinase. Activated Raf-1 then phosphorylates and activates MEK, which in turn phosphorylates and activates ERK. The activated ERK then moves to the nucleus to regulate the transcription of the targeted genes.

Here, we mainly focus on studying the subset of ERK signal transduction pathway regulated by RKIP. The considered biochemical reactions of the ERK pathway regulated by RKIP are as follows:

1. RKIP binds Raf-1* and forms a complex Raf-1*/RKIP;
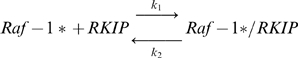
(3)
2. The activated ERK-PP interacts with the Raf-1*/RKIP complex to form a Raf-1*/RKIP/ERK-PP complex;
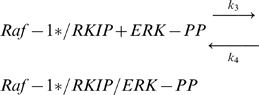
(4)
3. Phosphorylated RKIP-P, Dephosphorylated ERK and free Raf-1* are released from the complex Raf-1*/RKIP/ERK-PP;
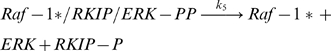
(5)
4. Double phosphorylated MEK activates ERK and forms MEK-PP/ERK complex;
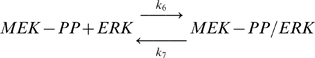
(6)
5. Dissociation of the complex MEK-PP/ERK and ERK phosphorylation;
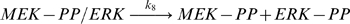
(7)
6. RP (RKIP-phosphotase) intacts with the RKIP to form complex RKIP-P/RP;
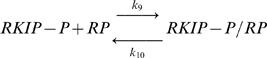
(8)
7. Disassociation of the complex RKIP-P/RP and dephosphorylation of RKIP-P;
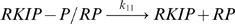
(9)

Let *x*_1_, *x*_2_, *x*_3_, *x*_4_, *x*_5_, *x*_6_, *x*_7_, *x*_8_, *x*_9_, *x*_10_ and *x*_11_ be the concentrations of Raf-1*, RKIP, Raf-1*/RKIP, Raf-1*/RKIP/ERK-PP, ERK, RKIP-P, MEK-PP, MEK-PP/ERK, ERK-PP, RP and RKIP-P/RP, respectively. We define the rates of reactions as follows:
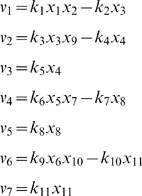
The stoichiometrix for the biochemical reaction of the Ras/Raf/MEK/ERK pathway is given by
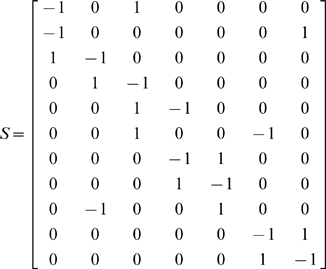
Let *x* = [*x*_1_, *x*_2_,…, *x*_11_]*^T^*, *V*(*x*) = [*v*_1_, *v*_2_,…, *v*_7_]*^T^*. The differential equations for the biochemical reactions of the Ras/Raf/MEK/ERK pathway is then given by
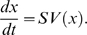
(10)


### Nonlinear State-Space Models

As shown in previous sections, the problems encountered in biochemical networks are of a nonlinear nature. Biochemical networks can be treated as nonlinear dynamic systems. A very powerful approach to deal with dynamic systems is the state-space approach [36]. To develop nonlinear state-space models for biochemical networks requires identifying the variables, their components and biochemical reactions which characterize dynamics of the biochemical networks. In most case, variables in biochemical networks are concentrations of metabolites, enzyme and proteins or gene expression levels. In general, these variables can be divided into three types of variables: input, output and state variables. State variables making up the smallest variables that determine the state of dynamic system are often not easily accessible for measurements (hidden), but essential for evolution of the system over time. Output variables are observed variables, which are functions of state variables.

The nonlinear state-space model is defined by two types of equations: state equations that define the dynamics of biochemical networks through time and observation equations that describe how the state variables are observed. The popular state equations for dynamics of biochemical networks are defined by dynamic mass balance equations or kinetic models. As we illustrated in the previous section, the general kinetic models can be formulated as [37]:
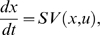
(11a)

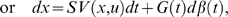
(11b)where S is a stoichiometrix matrix that describes the biochemical transformation in a biochemical network, *x* is a vector of state variables which are concentrations of metabolites, enzyme and proteins or gene expression levels, *u* is a vector of input variables, and *V*(*x*, *u*) is the vector of reaction rates and is usually the vector of nonlinear function of the state and input variables, *G*(*t*) is the dispersion matrix, *β*(*t*) is a Brownian motion with diffusion matrix Q(t) [38]. Equation (11a) is ordinary differential equations, and equation (11b) is stochastic differential equations. Intuitively, equation (11b) can be considered as the ordinary differential equation (11a) driven by random white noise processes w (t) as follows
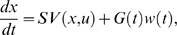
where 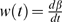 is a Gaussian white noise process in the sense that *w*(*t*) and *w*(*s*) are uncorrelated (and independent) for all *t*. Stochastic differential equations can incorporate the system noise into the model. Equation (11a) or equation (11b) determines the evolution of biochemical networks. Thus, it is often referred to as state equation or system equation.

Let *y* be a vector of observed or output variables. The observation equation that defines the relationships between the observed variables, and state-input variables is given by
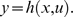
(12)Equation (12) does not consider noise. However, measurement noise always exists in biochemical systems. The noise should be incorporated into the models. Equation (11a) is a continuous ordinary differential equation and equation (11b) is a continuous stochastic differential equation. Many estimation methods are based on discrete-time dynamic systems. Thus, equation (11) needs to be changed into difference equation. A general discrete nonlinear model for biochemical networks is given by
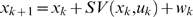
(13)In more general, the nonlinear state-space model for biochemical networks is given by
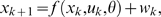
(14)where, *x_k_* is an *m*-dimensional vector of state variables, *u_k_* is an *l*-dimensional vector of input variables, *f* is an *m*-dimensional vector of nonlinear functions, *w_k_* denotes zero-mean uncorrelated Gaussian noise with covariance matrix *Q_k_*. The *p*-dimensional vector of measurements *y_k_* is related to the unobserved hidden state variable through the observation equation:
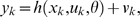
(15)Where, *h* is a *p*-dimensional vector of nonlinear functions and *v_k_* is uncorrelated Gaussian noise with covariance matrix *R_k_*. We assume that the random processes *w_k_* and *v_k_* are mutually independent. The initial state *x*_0_ is assumed to be Gaussian distributed with mean *a*_0_ and covariance matrix *P*_0_. We also assume that the vector of parameters *θ* is identifiable.

### Extended Kalman Filter (EKF) for Dual Estimation

The challenging tasks in inference for nonlinear state-space models are to estimate both the states and parameters of the systems from input variables and noise observations. One of methods for this dual estimation is to use EKF by taking the parameters as additional states and augmenting state equations [39]. Let *Z* = [*x^T^*, *θ^T^*]*^T^*. The augmented state equations are given by
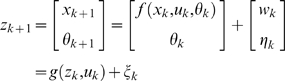
(16)Where, *η_k_* is uncorrelated Gaussian noise with covariance matrix Φ*_k_*. After extending the state variables with the parameter vector, the observation equation becomes
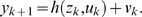
(17)The basic idea behind the Kalman filter is that it operates by propagating the mean and covariance of the state through time [22].

Define
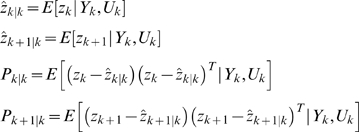
Where, 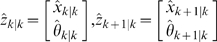.

Let
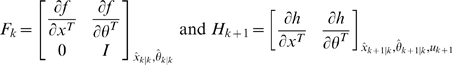
(18)Then, the EKF algorithm for dual estimation consists of two steps: prediction and filtering:

Prediction:

1. Given previous estimated state *zˆ* *_k_*_|*k*_, the observation *Y_k_* and new input *u_k_*, the system then moves to a new state. We attempt to predict the new state of system at time *t_k_*_+1_:
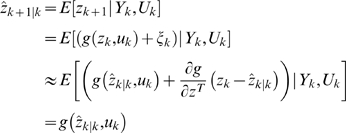
(19)

The variance matrix of prediction error can be calculated as
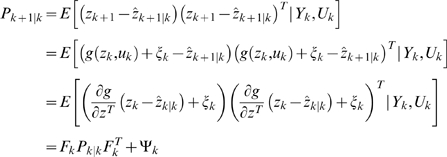
Where, *P_k_*_|*k*_ = *E*{[(*z_k_*−*zˆ* *_k_*_|*k*_)[(*z_k_*−*zˆ* *_k_*_|*k*_)]*^T^*} is the variance matrix of the filter error and 
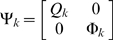
;

Filtering:

In the filtering cycle, we use the observation *y_k_*_+1_ at time *t_k_*_+1_ to update estimation of the state of system at time *t_k_*_+1_. In other words, in the filtering cycle, we attempt to improve the information on *z_k_*_+1_ after the new observation *y_k_*_+1_ is available. The error of the measurement prediction or innovation process is defined as
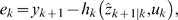
Where, the error includes the novelty or the new information which is contained in the new observation. The estimator of the state of system at time *t_k_*_+1_ is given by
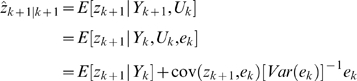
After some calculation, we obtain the update of the state estimation:

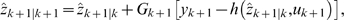
Where, *G_k_*_+1_ is the Kalman gain matrix defined as

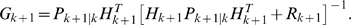
The filtered state estimate is the summation of the predicted state estimate and gain state error which the new observation *y_k_*_+1_ brings. The updated estimate covariance matrix is given by

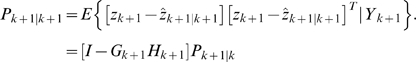
(20)

## Results

To evaluate its performance for estimation of parameters in nonlinear state-space model of biochemical networks, the EKF was applied to simulation data, the real experimental data of the JAK-STAT pathway and Ras/Raf/MEK/ERK pathway.

### Simulated Data

**Figure 3:**
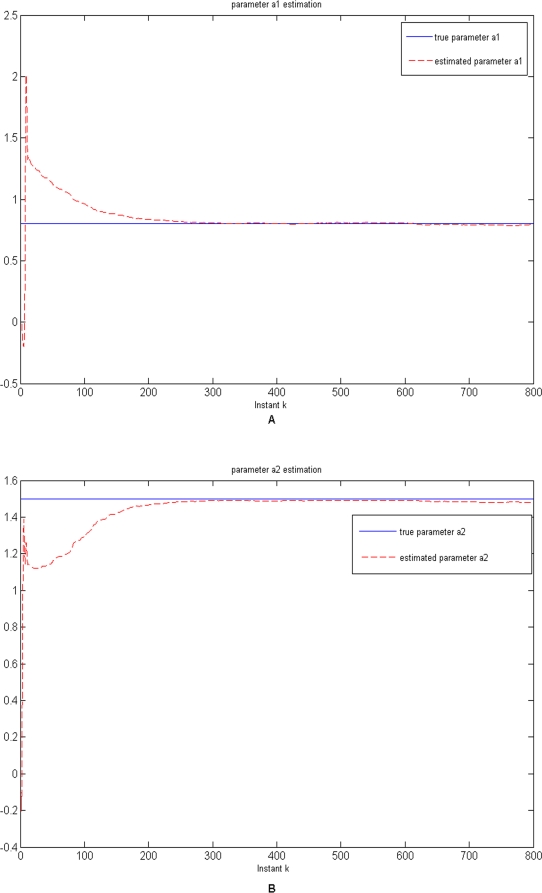
3A. The estimated parameter *θ*_1_ for the simulated data. 3B. The estimated parameter *θ*_2_ for the simulated data.

The data were simulated according to the following discrete nonlinear model [40]:
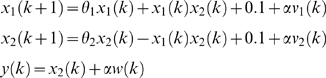
where, *θ*_1_ = 0.8, *θ*_2_ = 1.5, *α* = 0.01, the independent zero-mean noise *v*_1_, *v*_2_, *w* which obey the following discrete distributions:
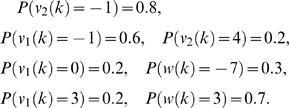
Simulations were performed for 800 equally spaced time points. The estimation process began with the initial values of the state variables *x*_0_ = [1.35,0.11]*^T^*. The initial values of the parameters were assigned to zero, i. e. *θ*_0_ = [0,0]*^T^*. The estimated parameters as a function of time *k* were shown in Figures 3A and 3B, where the solid lines were true parameters. From Figures 3A and 3B we can see that at the beginning the estimated parameters show fluctuations, but they quickly converge to the true parameters. This example demonstrated that although the parameters were treated as the states of the systems and hence may change over time, they can reach stable values. From Figures 3A and 3B we also can see some limitations on the Kalman filter for less than 100 data points. One way to overcome the limitations is to choose appropriate covariance matrix Φ*_k_* of the noise in the parameter equation (16). It is well known that the covariance matrix Φ*_k_* will affect the convergence rate and tracking performance [41]. A simple way to chose Φ*_k_* is to set Φ*_k_* to an arbitrary value, and make this towards zeros as the EKF proceeds.

### The JAK-STAT Pathway

The time-course experiments were performed four times for the core module of the JAK-STAT pathway, which was shown in Figure 1 [2], [33]. For each time point, 10^7^ cells were taken from the pool of BaF3cells. The state variables include concentrations of unphosphorylated STAT5 (*x*_1_), tyrosine phosphorylated monomeric STAT5 (*x*_2_), tyrosine phosphorylated dimeric STAT5 (*x*_3_) and nuclear STAT5 (*x*_4_). Unfortunately, to experimentally measure all individual STAT5 is difficult. Only concentrations of tyrosine phosphorylated STAT5 in the cytoplasm and total STAT5 in the cytoplasm were measured at 16 time points (from 0 to 60 minutes) by quantitative immunoblotting. In addition, measurements of Epo-induced tyrosine phosphorylation EpoR (EpoR_A_) as input were available.

**Figure 4:**
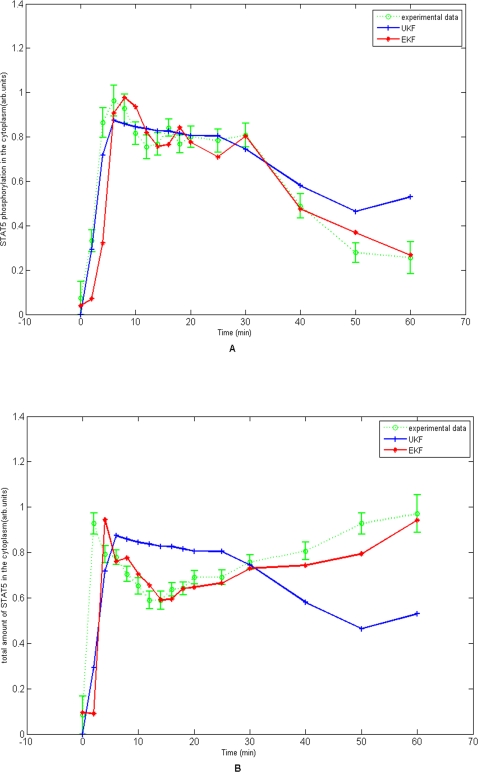
4A. The predicted and observed concentrations of tyrosine phosphorylated STAT5 in the cytoplasm for experiment 1. 4B. The predicted and observed concentrations of total STAT5 in the cytoplasm for experiment 1.

**Figure 5:**
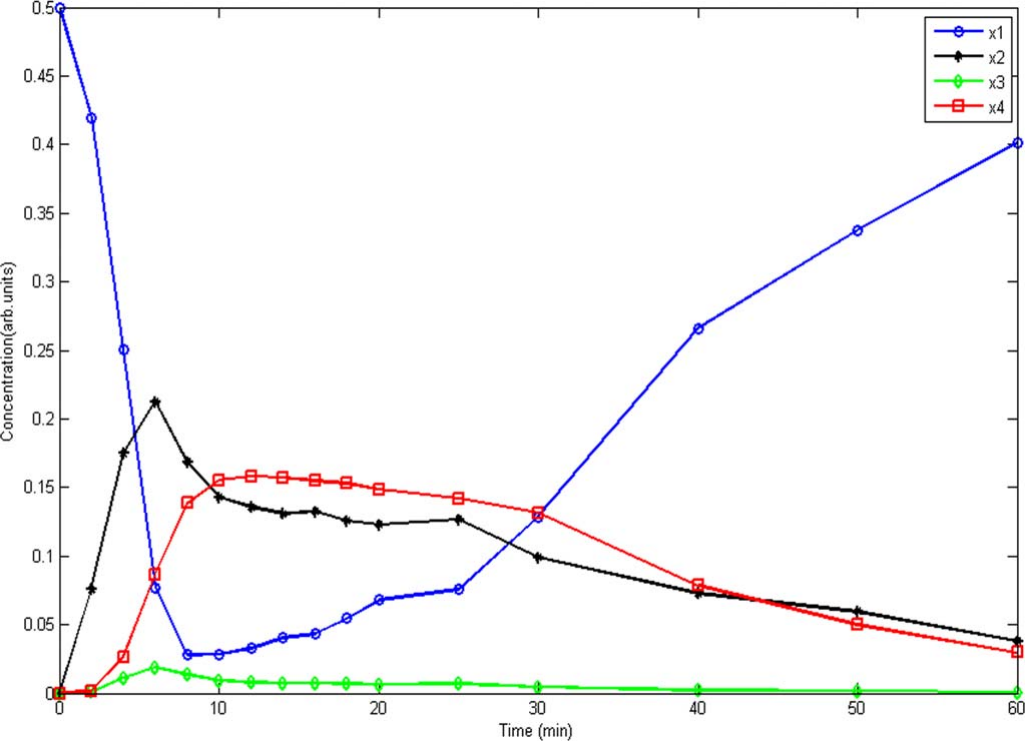
The predicted dynamic behavior of unphosphorylated STAT5 (*x*_1_), tyrosine phosphorylated STAT5 monomers (*x*_2_) and dimers (*x*_3_) in the cytoplasm, and STAT5 molecules in the nucleus (*x*_4_) in the JAK-STAT pathway.

The initial values of the state variables and parameters were assumed as *x*_1_ = 0.1, *x*_2_ = 0, *x*_3_ = 0, *x*_4_ = 0, *k*_1_ = 0.017, *k*_2_ = 2.1768, *k*_3_ = 0.1184 and *k*_4_ = 0.1. The estimated parameters were listed in Table 1. The estimates by the EKF and maximum likelihood approach [2] were close, but significantly different from the estimates by unscented Kalman filter (UKF) [19]. Using the estimated parameters and the concentration of EpoR_A_ as input, given initial values *x*_1_ = 0.1, *x*_2_ = *x*_3_ = *x*_4_ = 0, we can predict evolution of the state variables and observed concentrations of tyrosine phosphorylated STAT5 in the cytoplasm and total STAT5 in the cytoplasm. Figures 4A and 4B plot the predicted by the EKF and the UKF and observed concentrations of tyrosine phosphorylated STAT5 in the cytoplasm and total STAT5 in the cytoplasm (*y*_1_ and *y*_2_) in which all time-course data from four experiments were used to estimate parameters and observed data were from experiment 1. From Figures 4A and 4B we can see that the model fits the data very well. Figures 4A and 4B also demonstrated that the EKF fitted the concentrations of tyrosine phosphorylated STAT5 in the cytoplasm better than the UKF when the time passed 30 minutes and that the EKF fitted total STAT5 in the cytoplasm much better than the UKF for the most time points. Figure 5 showed the predicted dynamic behavior of unphosphorylated STAT5 (*x*_1_), tyrosine phosphorylated STAT5 monomers (*x*_2_) and dimers (*x*_3_) in the cytoplasm, and STAT5 molecules in the nucleus (*x*_4_).

**Table 1:** Estimated Parameters in the Nonlinear State-Space Model for the JAK-STAT Pathway.

Study	*k*_1_	*k*_2_	*k*_3_	*k*_4_	*τ*
Our Study	0.0211	2.2788	0.1064	0.1057	6 min
Swameye et al. (2003)	0.0210	2.4600	0.1066	0.1066	6.4 min
Wuach et al.( 2007)	0.0515		3.3900	0.3500	

### Ras/Raf/MEK/ERK Pathway

**Figure 6:**
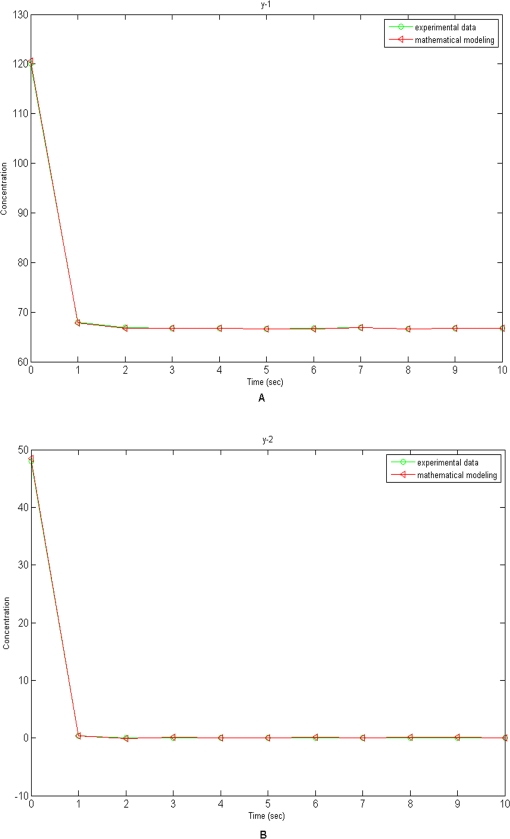
6A. The observed and predicted concentrations of Raf-1* in the Ras/Raf/MEK/ERK Pathway. 6B. The observed and predicted concentrations of RKIP in the Ras/Raf/MEK/ERK Pathway.

To investigate the impact of RKIP on the dynamics of the ERK pathway, an experiment was conducted [42]. The concentrations of Raf-1*, RKIP, Raf-1*/RKIP, Raf-1*/RKIP/ERK-PP, ERK, RKIP-P, MEK-PP, MEK-PP/ERK, ERK-PP, RP and RKIP-P/RP at ten equally spaced time points were collected. Since the EKF takes parameters as the state variables, the estimated parameters may vary over time. However, in the model we assume that the parameters are constants. The reasonable estimates of the parameters should reach to steady-state values. The steady-state values of the estimated parameters were summarized in Table 2 where we also listed the estimated parameters obtained by solving difference-algebraic equations [42]. Table 2 demonstrated that both estimates of the parameters were very close. To compare discrepancy between the estimated and observed concentrations of proteins, we performed simulations using the nonlinear state-space model given by equation (10) and estimated parameters. In simulations, the initial values of the states and parameters were assumed in Table 3. We plotted Figures 6A and 6B showing the observed and predicted concentrations of Raf-1* and RKIP as a function of time. We can see from Figures 6A and 6B that the model quite accurately predicted the concentrations of Raf-1* and RKIP.

**Table 2:** The estimates of the parameters in nonlinear state-space model of ERK pathway.

Parameters	Estimates by EKF	Estimates by Cho et al. (2003)
k_1_	0.5242	0.5300
k_2_	0.0075	0.0072
k_3_	0.6108	0.6250
k_4_	0.0025	0.00245
k_5_	0.0371	0.0315
k_6_	0.8101	0.8000
k_7_	0.0713	0.0075
k_8_	0.0687	0.0710
k_9_	0.9600	0.9200
k_10_	0.0012	0.00122
k_11_	0.8720	0.8700

**Table 3:** The initial values of the concentrations of the proteins and parameters.

*x*_1_	*x*_2_	*x*_3_	*x*_4_	*x*_5_	*x*_6_	*x*_7_	*x*_8_	*x*_9_	*x*_10_	*x*_11_
66	0.054	0.019	59	0.09	0.012	65	26	175	161	2.18
*k*_1_	*k*_2_	*k*_3_	*k*_4_	*k*_5_	*k*_6_	*k*_7_	*k*_8_	*k*_9_	*k*_10_	*k*_11_
0.546	0.014	0.619	0.046	−1.29	0.84	−0.05	0.43	0.98	−0.006	0.88

## Discussion

Biochemical pathways form an intricate network of functional and physical interactions between molecular species in the cell. To understand system behavior of biochemical pathways requires developing mathematic models of biochemical networks. In this report, we addressed two important issues for modeling biochemical networks. One issue is to develop a general framework for modeling biochemical networks. Second issue is how to estimate the parameters in the models.

Kinetic models have been widely used mechanistic models for biochemical networks and hence should be the basis of mathematic models of biochemical networks. However, pure kinetic models for investigation of biochemical networks have limitations. First, deterministic kinetic models do not incorporate systems noise, which widely exist in the biochemical networks, into their formulations of biochemical networks. Second, only a rather small portion of noise corrupted observations of metabolites and proteins is available. Many quantities that determine the states of biological systems cannot be directly measured [43]. For example, gene regulatory systems involve a number of known and unknown biological machinery such as transcription factors, microRNA, chromatin, and biochemical modifications, which regulate the expression of the genes [44]. Neither activity level of regulator protein nor most of the upstream biochemical events regulating the activity of proteins can be measured today [45]. To overcome these limitations, a kinetic equation-based nonlinear state-space model was taken as a general framework for modeling biochemical networks in this report.

Kinetic models provide mechanisms for description of biochemical networks. We took the recent formulation of kinetic models for biochemical networks in which the derivatives of the concentrations of the compounds in the network are decomposed into the product of the stoichiometrix matrix and vector of the reaction rates [37]. We then extended the kinetic model of the biochemical network to including system noises. The extended kinetic equation was used as a system equation in the nonlinear state-space model.

To deal with a large number of unmeasured quantities in the biochemical reactions, we added observation equations that incorporate the unmeasured states and the observed quantities into the model. In the report, we demonstrated that the presented nonlinear state-space models for biochemical networks that consist of systems and observation equations not only can deal with both hidden and observed variables, but also can cover both deterministic and random variables. The nonlinear state-space models provide a very general framework for modeling a wide range of biological systems [19].

Parameter estimation is another key issue for modeling biochemical networks. Efficient parameter estimation methods should share a common feature which can handle the noises due to both systems and measurements. A common principle for most of current methods for estimation of parameters in the models of biochemical networks is to minimize description between the observed and predicted quantities. Therefore, these methods cannot handle systems noise, often reach a local optimum, and require intensive computations. In engineering, widely used methods for parameter estimation in nonlinear models of dynamic systems are to jointly estimate the states of the systems and parameters in the model. Recently, Quach and his coworkers [19] proposed to use the unscented Kalman filter (UKF) to estimate the parameters in the nonlinear state-space model of biochemical network. The UKF is the recently developed method to simultaneously estimate the states of the system and parameters in the model. Alternative to the UKF, in this report, we proposed to use the EKF for parameter estimation. The EKF is the widely used methods for estimation of both the states and parameters. The EKF is easy to implement and requires less computational time than other methods. Although, in general, the EKF is thought to be less accurate for parameter estimation in nonlinear dynamic systems, our preliminary results in the report showed that the EKF can also reach very good accuracy in estimation of nonlinear dynamic models of biochemical networks. In addition to the EKF and UKF for parameter estimation in the nonlinear models of dynamic systems, a number of new methods based on sequential Monte Carlo (SMC) methods and expectation-maximization methods for parameter estimations have been developed. All these methods are based on Kalman filter. We can expect that the Kalman filter-based parameter estimation methods for nonlinear dynamic models will open a new avenue for investigation of large-scale biochemical networks.

As previously discussed, in this report we considered two errors: the system or process noise and the measurement noise. When all systematic information about the studied network has been included in the models, there will be random effects which have not been incorporated into the model. Also, in practice, there are measurement errors. Therefore, in this report, we assumed that the process noise and measurement noise existed and that the process noise and measurement noise were both white. However, in practice, the noise may not be white. In this case, we need to consider colored process noise and measurement noise. We also assumed that the process noise and measurement noise were uncorrelated. In practice, the process noise and measurement noise may be correlated. Correlation between process and measurement noise should be considered.

We assumed that the variance matrices of the process noise and measurement noise were known. This assumption is not realistic and hence should be released. The procedures for estimation of the variance matrices of the noise should be incorporated into the EKF in the future.

The Kalman filter can be either viewed as a minimum mean square estimates or a maximum posterior estimates. The EKF can also be interpreted as maximum likelihood estimate if we assume that the system noise and measurement errors follow Gaussian processes [46]. In general, the EKF methods may obtain only local optimum rather than global optimal solutions. A heuristic approach to sidestepping the multi-mode problem is to start algorithms many times by randomly selecting initial values for the states and parameters.

The size of the network which the EKF can fit depends on the number of time points and the number of replications (number of samples). Due to the experimental cost of measuring kinetic data, the number of time points and replications are often limited, which will affect the size of the network the EKF can fit. Also, estimation of the covariance matrices *Q_k_* and *R_k_* will increase the number of parameters and hence may affect the size of the network the EKF can fit. Since it takes the parameters as the states, the EKF increases the number of state variables. In this report, we have not studied whether this will reduce the size of the network the EKF fits. Simulations to address this issue should be carried out in the future.

Unlike the maximum likelihood estimate or EM algorithms where the initial values of the states and their covariance matrix can be optimally estimated, a quite open subject for the dual EKF is the choice of the initial values for both states and parameters. To avoid complexity, in this report we used a trial-and-error procedure to estimate the initial values for getting the tradeoff between global optimality of the estimators and convergence. However, selection of the initial parameters is important in ensuring convergence of the EKF algorithm. Estimation of initial values of both states and parameters should be incorporated into the EKF algorithms in the future.

One limitation of this report is that standard errors on parameters and an error variance to measure fit have not been estimated. Although the results of distributions of the estimators of the parameters in the nonlinear state space models in the literature have been limited, we will investigate asymptotical distributions of the estimators of the parameters and estimate the standard errors on parameters as well as the error variance to measure fitness by resampling methods in the future.
